# Terahertz flexible multiplexing chip enabled by synthetic topological phase transitions

**DOI:** 10.1093/nsr/nwae116

**Published:** 2024-03-23

**Authors:** Hang Ren, Su Xu, Zhidong Lyu, Yuanzhen Li, Zuomin Yang, Quan Xu, Yong-Sen Yu, Yanfeng Li, Fei Gao, Xianbin Yu, Jiaguang Han, Qi-Dai Chen, Hong-Bo Sun

**Affiliations:** State Key Laboratory of Integrated Optoelectronics, College of Electronic Science and Engineering, Jilin University, Changchun 130012, China; State Key Laboratory of Integrated Optoelectronics, College of Electronic Science and Engineering, Jilin University, Changchun 130012, China; College of Information Science and Electronic Engineering, Zhejiang University, Hangzhou 310027, China; College of Information Science and Electronic Engineering, Zhejiang University, Hangzhou 310027, China; College of Information Science and Electronic Engineering, Zhejiang University, Hangzhou 310027, China; Center for Terahertz Waves and College of Precision Instrument and Optoelectronics Engineering, Key Laboratory of Optoelectronic Information Technology (Ministry of Education of China), Tianjin University, Tianjin 300072, China; State Key Laboratory of Integrated Optoelectronics, College of Electronic Science and Engineering, Jilin University, Changchun 130012, China; Center for Terahertz Waves and College of Precision Instrument and Optoelectronics Engineering, Key Laboratory of Optoelectronic Information Technology (Ministry of Education of China), Tianjin University, Tianjin 300072, China; College of Information Science and Electronic Engineering, Zhejiang University, Hangzhou 310027, China; College of Information Science and Electronic Engineering, Zhejiang University, Hangzhou 310027, China; Center for Terahertz Waves and College of Precision Instrument and Optoelectronics Engineering, Key Laboratory of Optoelectronic Information Technology (Ministry of Education of China), Tianjin University, Tianjin 300072, China; Guangxi Key Laboratory of Optoelectronic Information Processing, School of Optoelectronic Engineering, Guilin University of Electronic Technology, Guilin 541004, China; State Key Laboratory of Integrated Optoelectronics, College of Electronic Science and Engineering, Jilin University, Changchun 130012, China; State Key Laboratory of Integrated Optoelectronics, College of Electronic Science and Engineering, Jilin University, Changchun 130012, China; State Key Laboratory of Precision Measurement Technology and Instruments, Department of Precision Instrument, Tsinghua University, Beijing 100084, China

**Keywords:** terahertz technology, topological phase transitions, flexible multiplexing, 6G communications, silicon chips

## Abstract

Flexible multiplexing chips that permit reconfigurable multidimensional channel utilization are indispensable for revolutionary 6G terahertz communications, but the insufficient manipulation capability of terahertz waves prevents their practical implementation. Herein, we propose the first experimental demonstration of a flexible multiplexing chip for terahertz communication by revealing the unique mechanism of topological phase (TP) transition and perseveration in a heterogeneously coupled bilayer valley Hall topological photonic system. The synthetic and individual TPs operated in the coupled and decoupled states enable controllable on-chip modular TP transitions and subchannel switching. Two time-frequency interleaved subchannels support 10- and 12-Gbit/s QAM-16 high-speed data streams along corresponding paths over carriers of 120 and 130 GHz with 2.5- and 3-GHz bandwidths, respectively. This work unlocks interlayer heterogeneous TPs for inspiring ingenious on-chip terahertz-wave regulation, allowing functionality-reconfigurable, compactly integrated and CMOS-compatible chips.

## Teaser

Exploration and utilization of the phase transition mechanism in the bilayer topological photonic systems enable terahertz flexible multiplexing photonic chips on an all-silicon platform.

## INTRODUCTION

Globalized interconnection, the metaverse and intelligent society boost advanced wireless communication to terahertz bands (e.g. 110–170 GHz [1] and 252–322 GHz [2]) that possess abundant spectral resources for accommodating the exponentially expanding information transfer demand [1–7]. Recent advancements with regard to the chips for terahertz communications, such as light sources [8], polarizers [9], phase shifters [10], clocks [11] and programmable holographs [12], have all attracted a steadily increasing quantity of interest. In particular, multiplexing chips are indispensable devices for guaranteeing efficient, high-transmission data rates in advanced communication systems [13–21]. To date, terahertz multiplexing chips are typically limited to a specific physical domain (such as the frequency domain) and lack the ability to be reconfigured over a broad frequency range. By extending the multiplexed domain to higher dimensions, a flexible multiplexing chip that interleaves information sequences with on-demand temporal durations and spectral widths into the terahertz resource ocean will be highly desirable for the dynamic massive-user access of sixth-generation (6G) communication [22–25]. In addition to this flexible multiplexing, low crosstalk, compact dimension and environmentally favourable energy cost levels are all crucial for terahertz integrated communication systems [26]. However, the deficient capability of on-chip terahertz-wave manipulation poses a hindrance to the development of terahertz flexible multiplexing chips, not to mention simultaneously enabling the aforementioned crucial properties.

Recently developed topological photonics [27–29], which can involve valleys [30,31] and pseudospins [32,33], bring new possibilities for exotic on-chip wave manipulation. By constructing topologically protected propagating modes with the use of an in-plane topological phase (TP) transition, various novel devices, including defect-tolerant waveguides [34,35], compactly integrated lasers [36,37], topological antennas [38,39], sensors [40] and one-way fibres [41], have been developed in compact geometries. Recent studies on the in-plane TP transition, demonstrated in microwave [42,43] and optical [44] ranges, exhibit the potential for manipulating electromagnetic waves and light in reconfigurable systems. In 2022, it was demonstrated that reconfigurable photonic topological insulators could potentially be utilized in 5G wireless systems [45]. For 6G terahertz communications, valley Hall topological silicon photonics achieve non-adjustable waveguides and resonant demultiplexers as pioneers. These previous efforts have demonstrated the revolutionary potential of topological photonics for on-chip wave manipulation; however, the realization of terahertz flexible multiplexing photonic chips with reconfigurable channels will need further exploration [22].

**Figure 1.**
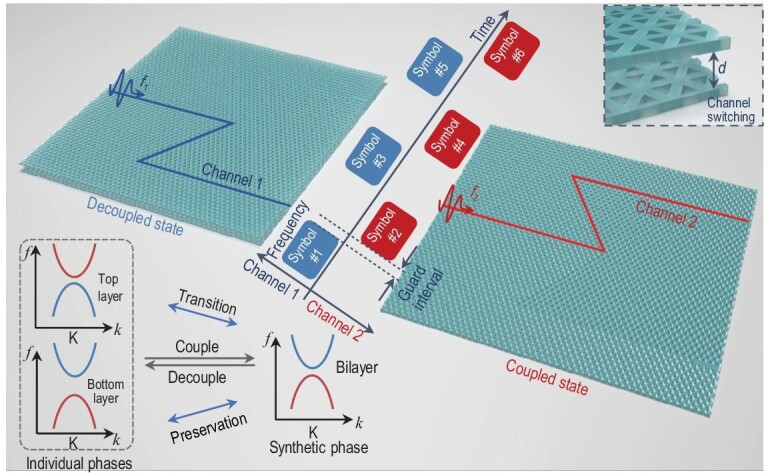
Schematic view of the flexible multiplexing chip. The subchannel switching is operated at the cost of a temporal guard frame determined by the switching time of interlayer distance *d*. The information is multiplexed and transported to corresponding ports on time-frequency interleaved channels, and its architecture is mentioned in several standard documents and proposals [22,23]. The insets show the simplified diagram of the energy bands with a TP transition mechanism.

In this article, we reveal the unique mechanism of on-chip wave manipulation enabled by previously unidentified synthetic TP transitions in a heterogeneously coupled valley Hall topological system, and propose the first experimental demonstration of a terahertz flexible multiplexing photonic chip (Fig. 1). The all-silicon bilayer topological system operates in a time-frequency interleaved complex domain. The strong coupling between the individual TPs of the two heterogeneous topological insulators generates a synthetic TP with a full band gap. By adjusting the interlayer distance *d* along the out-of-plane dimension, it is possible to transition between individual TPs in the decoupled state and synthetic TPs in the coupled state. The individual TP with a wide band gap is preserved during the coupling and decoupling processes, while a phase transition occurs between the individual TP with a narrow gap and the synthetic TP. This previously unidentified mechanism enables controllable terahertz on-chip modular TP transition and path switching. Two broadband frequency-division channels at ∼118–132 GHz are temporally switchable along their protected on-chip routines. We transmit and demultiplex 2.5-GHz-width 16-QAM signals to the corresponding outputs with speeds of 10 and 12 Gbit/s at carriers of 120 GHz and 130 GHz, respectively. The proposed on-chip flexible multiplexing architecture is consistent with several wireless communication standard documents and proposals [22,23]. In addition, 20-dB isolation is realized in a compact geometry on the topologically protected scattering-immune platform. The topological photonics-based all-silicon bilayer chip provides a compact, flexible and CMOS-compatible platform for upcoming 6G communications.

## RESULTS

### Synthetic bulk topological phases in bilayer systems

**Figure 2.**
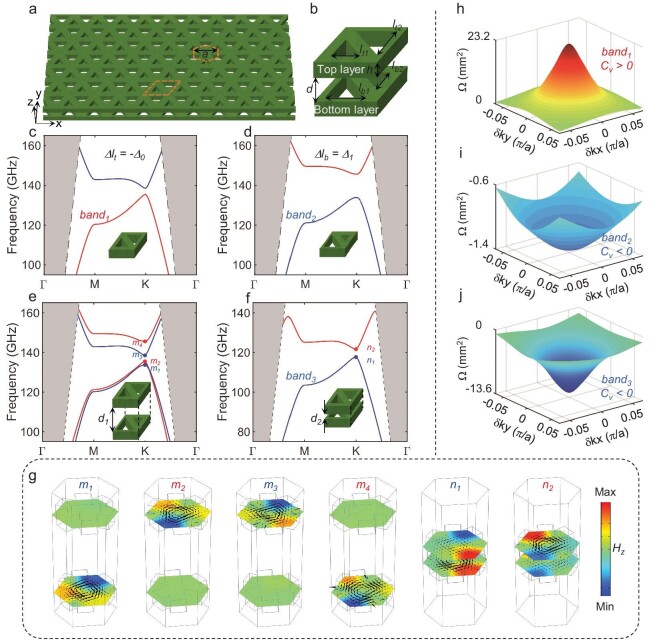
Synthetic bulk topological phases in a bilayer valley photonic crystal. (a, b) Schematic diagram of AA-stacked all-silicon bilayer VPC and its corresponding unit cell, respectively. (c, d) Band structures of individual top-layer and bottom-layer photonic crystals, respectively. (e, f) Band structures of bilayer VPC with interlayer distances of *d_1_* = 1000 μm and *d_2_* = 50 μm, respectively. Only the first and second synthetic bands are shown in (f). (g) The mode profiles of the K-valley eigenmodes *m_1-4_* and *n_1-2_* shown in (e) and (f). The black arrows indicate Poynting flow. (h–j) Berry curvatures near the K valley of the first band in (c), (d) and (f), respectively.

This study begins with the discovery of the TP transition and preservation mechanism in a bilayer valley photonic crystal (VPC) system made from silicon (${{\epsilon }_{{silicon}}}\ $= 11.7), as shown in Fig. 2a. The bilayer VPC consists of two layers of AA-stacked molybdenum disulfide-like photonic crystals with identical lattice constants *a* = 750 μm and thicknesses *t_silicon_* = 200 μm. The physical property of the bilayer valley Hall topological slab, i.e. the Hamiltonian, is written as


\begin{eqnarray*}
H = {{H}_T} + {{H}_B} + {{H}_{TB}},
\end{eqnarray*}

where *H_T_* is the top-layer Hamiltonian, *H_B_* is the bottom-layer Hamiltonian and *H_TB_* is the interlayer Hamiltonian (see details in Supplementary Information S1). In this equation, the interlayer Hamiltonian *H_TB_* can be adjusted by interlayer coupling strength, which is related to the interlayer distance *d* in our work. Therefore, the bilayer valley Hall topological slabs fundamentally provide more choices on the tunability. The geometries of the unit cell in each layer are distinct and can be described with equilaterally triangular air holes: *l_t1_* = *l_t0_* + *Δl_t_, l_t2_* = *l_t0_* − *Δl_t_, l_b1_* = *l_b0_* + *Δl_b_* and *l_b2_* = *l_b0_* − *Δl_b_*, as shown in Fig. 2b. The biased geometrical parameters *Δl_t_* and *Δl_b_* always satisfy the condition *Δl_t_Δl_b_ <* 0. The non-zero values of *Δl_t_* and *Δl_b_* break the symmetry of the individual top-layer and bottom-layer VPCs in opposite directions, respectively. That is, the isolated top-layer and bottom-layer VPCs always exhibit distinct photonic valley Hall TPs. For example, the energy bands of the individual top-layer VPC and bottom-layer VPC are given in Fig. 2c and d, respectively, using the parameters *l_t0_* = *l_b0_* = 375 μm, *Δl_t_ = −Δ_0_* = *−*35 μm and *Δl_b_* = *Δ_1_* = 139 μm. In contrast to the narrow *Δl_t_*-related band gap of the top-layer VPC, a wide but opposite *Δl_b_*-related band gap is achieved in the bottom-layer VPC.

Then, the stacked case is considered in the bilayer VPC system (see analytical calculation in Information S1). When the interlayer coupling is substantially weaker than the intralayer coupling, e.g. *d* = *d_1_* = 1000 μm, the bilayer chip operates in the decoupled state. The first and fourth energy bands of the bilayer VPC (Fig. 2e) are identical to those of the individual bottom-layer VPC in Fig. 2d, while the second and third bands (Fig. 2e) are identical to those of the individual top-layer VPC in Fig. 2c. The mode profiles are calculated to support this phenomenon. As shown in Fig. 2g, the magnetic field distribution and Poynting flow at the K-valley eigenmodes *m_1−4_* of weak-coupled bilayer VPCs are consistent with those of corresponding monolayer VPCs. These phenomena indicate that the decoupled bilayer VPC system preserves all the topological properties of individual monolayer VPCs. These preserved topological properties still exist on the corresponding monolayer VPC independently.

Moreover, the bilayer chip operates in the coupled state when the interlayer coupling is comparable to the intralayer coupling, e.g. *d* = *d_2_* = 50 μm. By checking modes n_1_ and n_2_ in Fig. 2g, the lowest two energy bands of the bilayer VPC exhibit a direct band gap at the K point in momentum space (Fig. 2f), which resembles a monolayer VPC with a low-frequency band gap. Interestingly, the direction of Poynting flow and the pseudospin chirality of modes *n_1_* and *n_2_* are consistent with those of the bottom-layer VPC and opposite to those of the top-layer VPC. By further comparing the Berry curvatures of individual monolayer VPCs (Fig. 2h and i) with those of strongly coupled bilayer VPCs (Fig. 2j), two strongly coupled heterogeneous monolayer VPCs can synthesize a new non-trivial bulk valley Hall TP with a complete band gap, in which the topological property, e.g. valley Chern number (*C_v_*), is always consistent with the monolayer VPC with a wide band gap.

The following findings are derived from the above-mentioned observations. First, the individual TPs and the synthetic TP in the decoupled and coupled states can be switched by the interlayer distance *d.* Second, the synthetic TP with a full band gap is generated from the strong coupling between the individual TPs of the two heterogeneous topological insulators. Third, the individual TP with a wide band gap is preserved, whereas the phase transition occurs between the individual TP with a narrow band gap and the synthetic TP. Fourth, the band gap of the transited synthetic TP shifts in the frequency domain compared to individual TPs.

### Flexible multiplexing enabled by synthetic edge states

**Figure 3.**
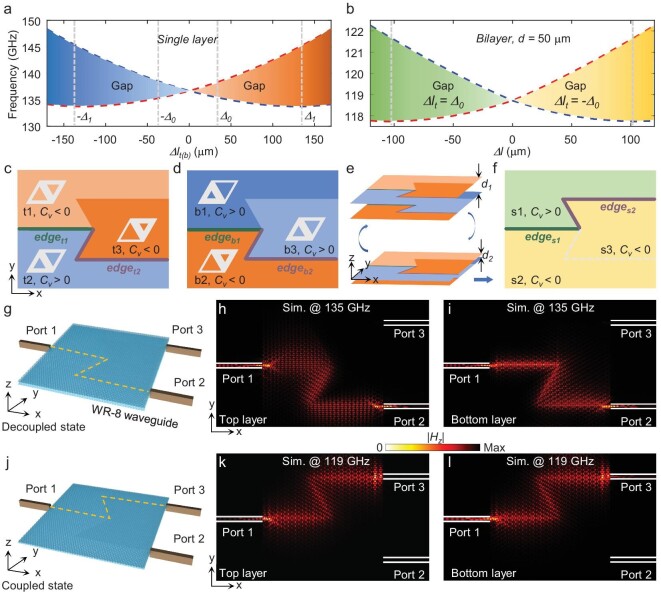
Reconfigurable topological edge state analysis. (a) Band gap of the individual top (bottom)-layer VPC as a function of *Δl_t__(b)_*. The distinct regions indicate distinct valley Hall topological phases for the decoupled state, respectively. (b) Band gap of the bilayer VPC as a function of *Δl* with *d* = 50 μm. The distinct regions indicate distinct valley Hall topological phases for the coupled state, respectively. The chrominance represents the size of the band gap. (c, d) Lattices and domain walls of the individual top and bottom layers. Without interlayer coupling, in-plane topological phase transitions occur at *edge_t1_* and *edge_t2_* of the top layer and *edge_b1_* and *edge_b2_* of the bottom layer. (e) Switching mechanism via interlayer coupling regulation. (f) Synthetic topological phases of the bilayer VPC at *d* = *d_2_* and the corresponding topological transition at *edge_s1_* and *edge_s2_*. (g, j) Full-wave simulation set-ups for decoupled and coupled cases, respectively. (h, i) Intensity distribution of *H_z_* on the top and bottom layers in the decoupled state, respectively. (k, l) Intensity distributions of *H_z_* on the top and bottom layers in the coupled state, respectively.

For a fundamental flexible multiplexing unit, two switchable subchannels with distinct on-chip routines have to be constructed over the time-frequency complex domain. According to our findings on the synthetic bulk TPs, the subchannels can be constructed with the use of topologically protected edge states, whereas the flexible switching capability over the time-frequency domain can be realized uniquely with the temporally transited and frequency-shifted synthetic TPs. Following this principle, we build a library of topological photonic properties of monolayer (decoupled state, Fig. 3a) and bilayer (coupled state, Fig. 3b) VPC systems. In the library, the width and frequency ranges of band gaps are determined by the symmetry-breaking geometric parameter *Δl_t__(b)_* for the monolayer VPCs and *Δl* = *Δl_t_* +* Δl_b_* for the bilayer VPCs.

Then, the bilayer VPC-based chip is designed by separating each monolayer VPC into three modules, as shown in Fig. 3c and d. For the top-layer VPC, Modules t1, t2 and t3 are obtained by selecting *Δl_t1_* = *Δ_0_, Δl_t2_* = −*Δ_0_* and *Δl_t3_* = *Δ_1_*, respectively; for the bottom-layer VPC, Modules b1, b2 and b3 are obtained by selecting *Δl_b,1_* = *Δ_1_, Δl_b,2_* = −*Δ_1_* and *Δl_b,3_* = *Δ_0_*, respectively. Here, the biased geometrical parameters, *Δ_0_ =* 139 μm and *Δ_1_ =* 35 μm, are obtained from Fig. 3a. Under this configuration, Module t1 and Module t3 have a negative *C_v_*, while Module t2 has a positive *C_v_*. Based on the bulk-edge correspondence, a topological valley kink edge state occurs along the boundaries e*dge_t1_* and *edge_t2_* between modules with opposite *C_v_* values in the individual top-layer VPC. Similarly, a topological valley kink edge state occurs along the boundary *edge_b1_* and *edge_b2_* in the individual bottom-layer VPC (see dispersion of the edge states in Information S2). Since the projections of boundaries *edge_t1−t2_* and *edge_b1−b2_* perfectly overlap on the chip surface (i.e. the x-y plane), a synthesized topological on-chip signal routine is realized from Port 1 to Port 2 in the weakly coupled bilayer VPC system (*d_1_* = 1000 μm, the upper part of Fig. 3e).

By decreasing *d* to *d_2_* = 50 μm (the lower portion of Fig. 3e), synthetic bulk valley Hall TPs are generated in all three modules due to the strong interlayer coupling. According to the synthetic principle of bilayer VPC systems, the topological properties of three bilayer strongly coupled modules inherit those of monolayer modules with an increased symmetry-breaking parameter *Δ_1_*. In this case, the *C_v_* values of synthetic valley Hall TPs are positive, negative and negative in modules s1, s2 and s3, respectively. As a result, a synthetic valley Hall edge state, i.e. *edge_s1_* and *edge_s2_*, forms as the other topological on-chip signal routine (Fig. 3f), i.e. from Port 1 to Port 3. Since the spectra of the topological on-chip routines are determined by the overlapped band-gap region of valley kink states, two distinct on-chip topological signal routines are locked to corresponding frequency-division channels. In addition, distance switching can be treated as a time-domain guide interval. Now, a time-frequency interleaved channel-switching architecture [22,23] is realized for terahertz flexible multiplexing chips, based on the unique properties of synthetic bulk TP transition and preservation.

For the proof of concept, a full-wave simulation of the flexible multiplexing chip with two broadband subchannels is performed before the fabrication of a realistic chip. As shown in Fig. 3g and j, the top- and bottom-layer VPCs of the chip are simultaneously coupled to the WR8 waveguides via tapered silicon pins. The chip is excited by the TE_10_-mode from Port 1. The out-of-plane magnetic field amplitude distributions |*H_z_*| within both layers of silicon structures at 135 GHz (*d* = 1000 μm, Fig. 3h and i) and 119 GHz (*d* = 50 μm, Fig. 3k and l) are plotted to show the on-chip routine. The frequency-division channels and signal-propagating routines, which are mediated by *d*, are freely switched on the chip. Moreover, the signals are immune from backscattering at sharp bends due to the existence of valley topological kink states.

### Demonstration of the flexible multiplexing chip

**Figure 4.**
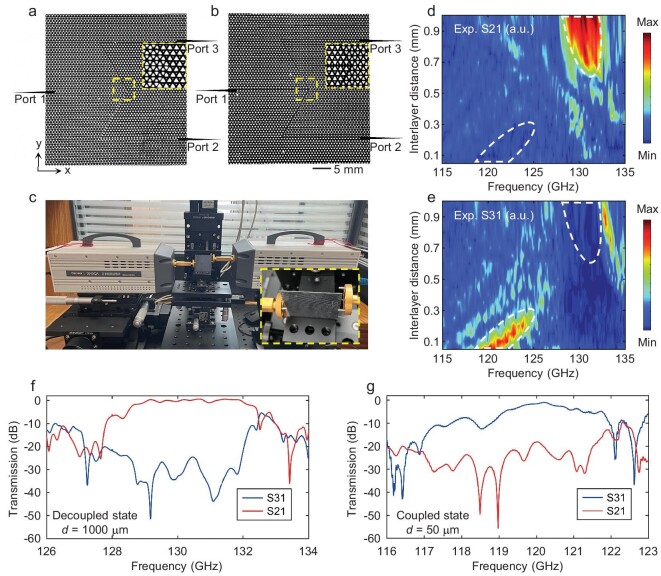
Experimental demonstration of the flexible multiplexing chip. (a, b) Optical images of the top- and bottom-layer photonic crystals of the chip, respectively. The insets show zoomed-in images of the photonic lattices. (c) Photograph of the test system. The inset shows the chip sample coupled with WR8 waveguides. (d, e) Measured intensities of S21 and S31 with the scanning interlayer distance *d*. The white dashed line marks the approximate range of the two switchable channels at the same location for comparison. (f, g) Normalized transmissions of the two frequency channels by using a monolayer straight topological waveguide (see details in Methods).

A realistic terahertz flexible multiplexing chip is fabricated by using high-resistance silicon wafers (ρ = 10 kΩ.cm) and commercial photolithography techniques. Figure 4a and b show the optical images of top- and bottom-layer VPCs, respectively. The size of the chip is ∼37.8 mm (*x*) by 40.9 mm (*y*), excluding the 10-mm-long tapered test pins. In the experiment, the working statuses of the bilayer VPC system are mechanically switched by varying *d* continuously within a range of 50∼1000 μm, as shown in Fig. 4c. The wave propagation and channel switching of the chip are verified by analysing the S parameters, e.g. S21 (Fig. 4d) and S31 (Fig. 4e). For the high-frequency-division channel (*d* > 700 μm), a high S21 is observed over the frequency range from 128 to 132 GHz at Port 2, while the low-frequency-division channel (*d <* 300 μm) propagates from Port 1 to Port 3 over the frequency range from 119 to 122 GHz. The whole testing process is shown in the supplementary video. Since the non-working channels are always associated with a bulk state at the photonic band gaps of VPCs, a 20-dB isolation is achieved between the working channel and the non-working channel, as shown in Fig. 4f and g.

### Terahertz high-speed on-chip communication

**Figure 5.**
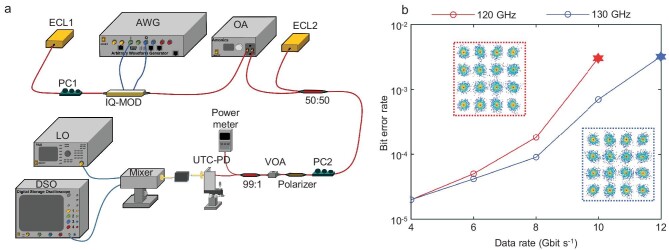
Terahertz information transmission using the flexible multiplexing chip. (a) Experimental set-up of the photonic transmission system carrying single-subchannel 2.5-GHz broadband 16-QAM signals. (b) Relationship between the transmission data rate and the BER achieved in the chip. The insets of (b) show the constellations of transmitted 16-QAM signals below the HD-FEC threshold marked by stars.

The terahertz communication of the flexible multiplexing chip is evaluated experimentally, and the set-up (see Methods) is shown in Fig. 5a. To match the practical communication standard, two 2.5-GHz and 3-GHz flat passbands of the switchable subchannels are selected to transmit the data streams in a 16-ary quadrature amplitude modulation (16-QAM) format around carrier frequencies of 120 and 130 GHz, respectively. The bit error rate (BER) as a function of data rate is employed to evaluate the communication performance, as shown in Fig. 5b. The communication data rate is considered when the measured BER values stay below the hard-decision forwards error correction (HD-FEC) threshold (i.e. 3.8 $\times \ $10^−3^), with 7% overhead. Under this threshold, 10 Gbit/s and 12 Gbit/s 16-QAM high-speed transmissions are achieved over the two subchannels. The spectral efficiency of the chip is 4 bit/s/Hz in both subchannels, reaching the theoretical limit of 16-QAM-modulated communications. The constellations shown in Fig. 5b indicate that the 16-QAM data streams can be demultiplexed and demodulated in our chip below the HD-FEC threshold for high-quality terahertz communication and flexible information multiplexed distribution. Therefore, the multiplexing functionality is demonstrated according to the demultiplexing experiment, since the valley topological kink states are reciprocal in the chip.

## CONCLUSION AND DISCUSSION

In conclusion, we reveal the intercoupling-dependent synthetic phase transition mechanism in heterogeneous bilayer valley Hall topological photonic crystals and build a reconfigurable on-chip signal manipulation architecture. As proof of concept, we experimentally demonstrate an all-silicon terahertz broadband flexible multiplexing chip with two broadband switchable frequency-division channels over ∼119–122 GHz and 128–132 GHz. In the experiments, we use the chip to transmit and demultiplex two streams of 16-QAM signals on interleaved time slots to distinct outputs with data rates of 10 and 12 Gbit/s around carriers of 120 GHz and 130 GHz under the HD-FEC threshold, respectively.

Notably, this work is the first experimental demonstration of switchable broadband on-chip multichannel photonic regulation over a time-frequency complex domain in terahertz bands. The synthetic TP can generate new topological edge states and enable the realization of terahertz flexible multiplexing photonic chips in an all-silicon platform. Benefiting from the reconfigurable channel architecture, flexible multiplexing technology can switch channels on demand in multi-user non-parallel scenarios, reducing crosstalk and saving channel resources, which is significantly different from existing terahertz frequency-division multiplexing [17–21,50]. The realized terahertz photonic flexible multiplexing conforms to the physical-layer architecture of cutting-edge wireless communications [20–25,46]. Our chip also features technological advancements in CMOS compatibility, high mutual-channel isolation and ultracompact integration. Furthermore, the mechanically driven photonic manipulation employed in our chip, which is free from the optoelectronic responses of natural materials and everlasting power supplies, may allow low-carbon emissions in future green information industries [26].

The synthetic valley Hall TP with a full band gap in the whole moment space below the light line is observed by utilizing the strong coupling between the individual TPs of two heterogeneous topological insulators, compensating the discoveries of previous studies [47–49]. Our findings on heterogeneous TPs contribute to a comprehensive understanding of topological insulators and may inspire unprecedented band engineering in photonics and condensed matter physics. For on-chip photonic regulation applications, the heterogeneous TP extends the monolayer architecture of silicon-based topological chips to the bilayer heterogeneous variety. This study advances topological insulators towards reconfigurable, compactly integrated and CMOS-compatible photonic chips, including matter-selective sensors, tuneable power dividers, beam-steering on-chip radiators and wavelength-selected emitters, for terahertz communications, intelligent earth and metaverse.

## METHODS

### Calculation and simulation

The band structures of the VPCs are obtained with a tight-binding model (see details in Information S5 and S6). The eigenmode simulation is conducted with Comsol Multiphysics. The time-domain full-wave simulation is conducted with CST Microwave Studio.

### Sample fabrication

The chip is fabricated with mask lithography and reaction ion etching. The thickness of the high-resistance (ρ = 10 kΩ·cm) silicon wafer is 200 μm.

### S-parameter measurement

The scattering parameters of the chip are measured with a vector network analyser (Ceyear 3672B) and two frequency multiplier modules (Ceyear 3643QA) over a spectral range from 90 to 140 GHz, as shown in Fig. 4a. Tapered waveguides are used to couple the signal from the port of WR8 rectangular metallic waveguides. A displacement platform (MTS203, Beijing Optical Century Instrument Co., Ltd.) is used to control *d* and spatially align bilayer VPCs. The step size and moving speed are 1 μm and 10 mm/s, respectively. The response time for channel switching is τ ≈ 100 ms and the maximum modulation speed is 5 Hz. The modulation depths are 18 dB and 21 dB for the S31 path and the S21 path, respectively (see details in Information S7). Since the majority of transmission loss is caused by test-purpose mode conversion, which can be eliminated in an all-VPC on-chip system, a monolayer straight topological waveguide consistent with *edge_t1_* is used to eliminate the loss caused by the mismatch. The transmission spectrums plotted in Fig. 4f and g are normalized by the max efficiency of the straight reference topological waveguide (−8.21 dB) (see details in Information S5 and S6).

### Channel measurement

Figure 5a illustrates the configuration employed for the measurement of channel characteristics. The emission of the optical continuous wave is generated by an external cavity laser (ECL1). Subsequently, the light wave is introduced into an in-phase (I) and quadrature (Q) modulator (IQ-MOD) controlled by an arbitrary waveform generator (AWG). To maximize the polarization of the incident light, a polarization controller (PC1) is utilized. Following amplification through an erbium-doped fibre amplifier (EDFA), the modulated optical carrier is merged with an optical local oscillator (LO) using a 3-dB optical coupler (OC). The terahertz communication signal is generated through photomixing in a uni-travelling carrier photodiode (UTC-PD), following polarization alignment by PC2 and a polarizer. A polarization-maintaining variable optical attenuator (VOA) is employed to regulate the input power of the UTC-PD to a level of −6 dBm. The terahertz signal is amplified using a power amplifier (PA) with a gain of 14 dB and is connected into the chip. Subsequently, the signal is gathered and subjected to downconversion within the intermediate frequency (IF) range. This process is facilitated by a Schottky mixer, which is activated by a 24-order electrical LO signal that has undergone frequency multiplication. The IF signal is directed to a digital storage oscilloscope (DSO) for conducting offline digital signal processing. This processing involves several techniques such as multi-modulus-algorithm (MMA)-based linear equalization, Viterbi & Viterbi frequency offset compensation and the blind phase search method.

## Supplementary Material

nwae116_Supplemental_File
