# Supplementary material for: Terahertz flexible multiplexing chip enabled by synthetic topological phase transitions
Source: Natl Sci Rev. 2024 Mar 23;11(8):nwae116. doi: 10.1093/nsr/nwae116 (PMC11242461; doi:10.1093/nsr/nwae116)
Supplement: nwae116_Supplemental_File [file nwae116_supplemental_file.zip › Supplementary Information.pdf]

Supplementary Information for

Terahertz Flexible Multiplexing Chip Enabled by Synthetic Topological Phase Transitions

Hang Ren[1,#], Su Xu[1,#,*], Zhidong Lyu[2,#], Yuanzhen Li[2], Zuomin Yang[2], Quan Xu[3], Yong-Sen Yu[1], Yanfeng Li[3], Fei Gao[2], Xianbin Yu[2,*], Jiaguang Han[3,4,*], Qi-Dai Chen[1], Hong-Bo Sun[1,5,*]

[1]State Key Laboratory of Integrated Optoelectronics, College of Electronic Science and Engineering, Jilin University, 2699 Qianjin Street, Changchun 130012, China.

[2]College of Information Science and Electronic Engineering, Zhejiang University, Hangzhou 310027, China.

[3]Center for Terahertz Waves and College of Precision Instrument and Optoelectronics Engineering, Key Laboratory of Optoelectronic Information Technology (Ministry of Education of China), Tianjin University, Tianjin 300072, China.

[4]Guangxi Key Laboratory of Optoelectronic Information Processing, School of Optoelectronic Engineering, Guilin University of Electronic Technology, Guilin 541004, China.

[5]State Key Laboratory of Precision Measurement Technology and Instruments, Department of Precision Instrument, Tsinghua University, Haidian, Beijing 100084, China.

#These authors contributed equally to this work.

*Author to whom correspondence should be addressed; E-mail: xusu@jlu.edu.cn (S. Xu), xyu@zju.edu.cn (X. Yu), jiaghan@tju.edu.cn (J. Han) and hbsun@tsinghua.edu.cn (H.-B. Sun).

S1. Tight-binding Hamiltonian for bilayer VPCs

We first simulate the photonic modes of the rhombus-shaped bilayer lattice with four complete air holes shown in Fig. S1(a). The white dotted line shows one of the four triangular resonators. Fig. S1(b) shows the four K-point eigenmodes *t1*, *t2*, *b1*, and *b2*. The mode profiles show that all the modes are confined in the resonators.

To clarify the role of interlayer coupling on the energy bands and the synthetic topological phase, we give the tight-binding Hamiltonian and energy bands of bilayer VPCs. As the photonic mode is confined in the resonator, the corresponding eigenfrequency is decided by the size of the air hole. The bilayer lattice shown in Fig. S1(a) can be translated into a tight-binding model shown in Fig. S1(c). Every single resonator, which has a confined photonic mode, couples with the others. The top-layer intralayer coupling coefficient, the bottom-layer intralayer coupling coefficient, and the interlayer coupling coefficient are described by κ_t, κ_b, and κ_{inter}, respectively. The Hamiltonian of this bilayer system is $H = H_T + H_B + H_{TB}$. The top-layer Hamiltonian H_T, bottom-layer Hamiltonian H_B, and interlayer Hamiltonian H_{TB} are expressed as:

$$
\begin{aligned}
H_T &= V_{T1} a_T^\dagger a_T + V_{T2} b_T^\dagger b_T + \kappa_T (a_T^\dagger b_T + b_T^\dagger a_T) \\
H_B &= V_{B1} a_B^\dagger a_B + V_{B2} b_B^\dagger b_B + \kappa_B (a_B^\dagger b_B + b_B^\dagger a_B) \\
H_{TB} &= \gamma_1 (a_T^\dagger a_B + a_B^\dagger a_T) + \gamma_2 (b_T^\dagger b_B + b_B^\dagger b_T) + \beta_1 (a_T^\dagger b_B + b_B^\dagger a_T) + \beta_2 (b_T^\dagger a_B + a_B^\dagger b_T)
\end{aligned}
$$

In these equations:

(1) $a_T, a_B, b_T,$ and b_B are Pauli matrices.

(2) $V_{T1}, V_{T2}, V_{B1},$ and V_{B2} represent the eigenfrequency of the resonant modes *t1*, *t2*, *b1*, and *b2*, respectively.

(3) γ_1 represents the coupling coefficient between *t1* and *b1*, γ_2 represents the coupling coefficient between *t2* and *b2*, β_1 represent the coupling coefficient between *t1* and *b2*, and β_2 represent the coupling coefficient between *t2* and *b1* [S1,S2].

Thus, the total interlayer coupling $\kappa_{inter} = (\gamma_1, \gamma_2, \beta_1, \beta_2)$. Here we approximate that, these four coupling coefficients change in the similar trend, when the interlayer distance changes. Then, the interlayer coupling strength can be described by a

simplified modulus length of κ_{inter}, instead of a complicated modulus tensor. Figs. S1(d) and (e) show the evolution of energy bands with interlayer coupling when $\Delta l_t + \Delta l_b = 0$ and $\Delta l_t + \Delta l_b \neq 0$, respectively. Interestingly, when the individual TPs have the same bandgap, the synthetic TPs exhibit Dirac degenerations (only the low-level mode is employed in the chip design). When we set the individual TPs with different sizes of bandgaps, this Dirac degeneration can be broken into a topological bandgap. These analytical results are consistent with the numerical and experimental results in the main text and can help to further understand the generation, transition, and disappearance of synthetic TPs.

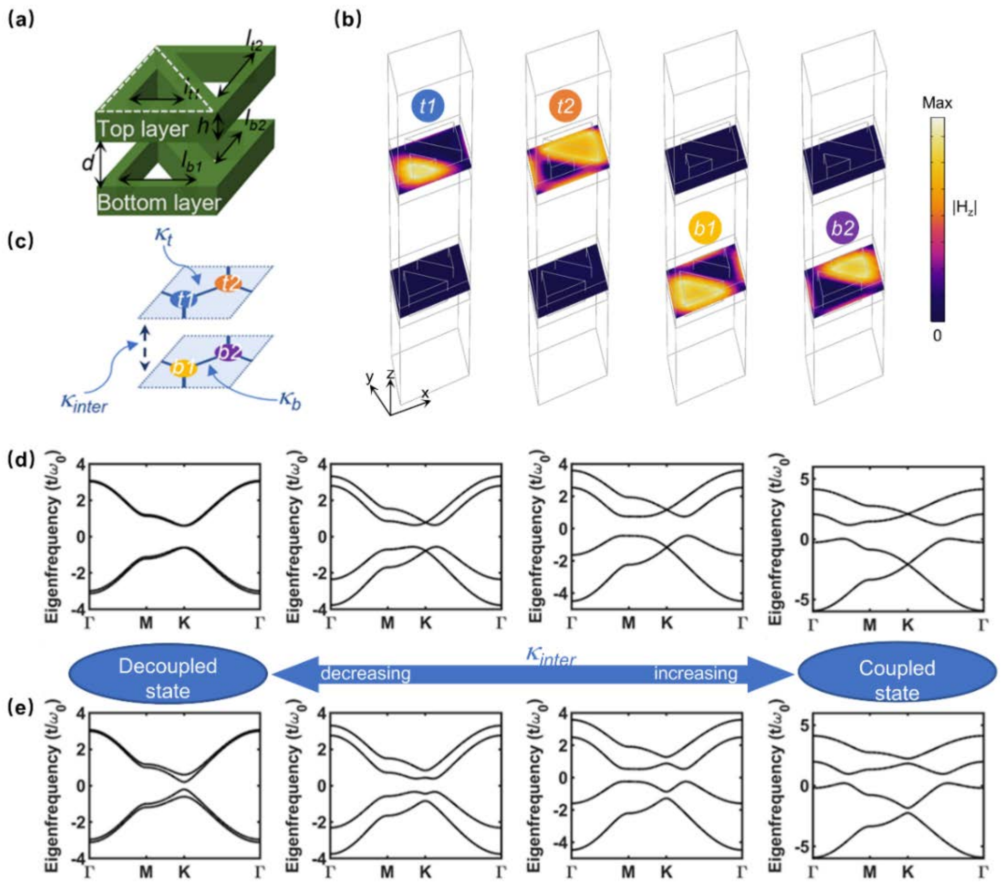

Figure S1. Tight-binding Hamiltonians for interlayer coupling-induced topological phase transitions. (a), The unit cell of the bilayer photonic lattice. The white dashed line shows an example of an effective resonator. (b), The eigenmodes of the four effective resonators at K point. (c), The tight-binding model of the four-resonator coupling system. (d, e), The evolution of the energy bands diagram with the variation of $\kappa_{inter.}$ (d) shows the case of $\Delta l_t + \Delta l_b = 0$ and (e) shows the case of $\Delta l_t + \Delta l_b \neq 0$.

S2. Dispersion of the reconfigurable edge states.

The dispersion of the edge states of the decoupled states and coupled states discussed in the main text Figs. 3c, 3d and 3f is calculated with numerical simulation. Figs. S2(a)-(f) show the dispersion of the valley kink states at $edge_{t1,2}$, $edge_{b1,2}$, and $edge_{s1,2}$. The inserts show the intensities of out-of-plane magnetic near the domain walls. The solid curves represent valley kink states considered in subsequent simulations and experiments.

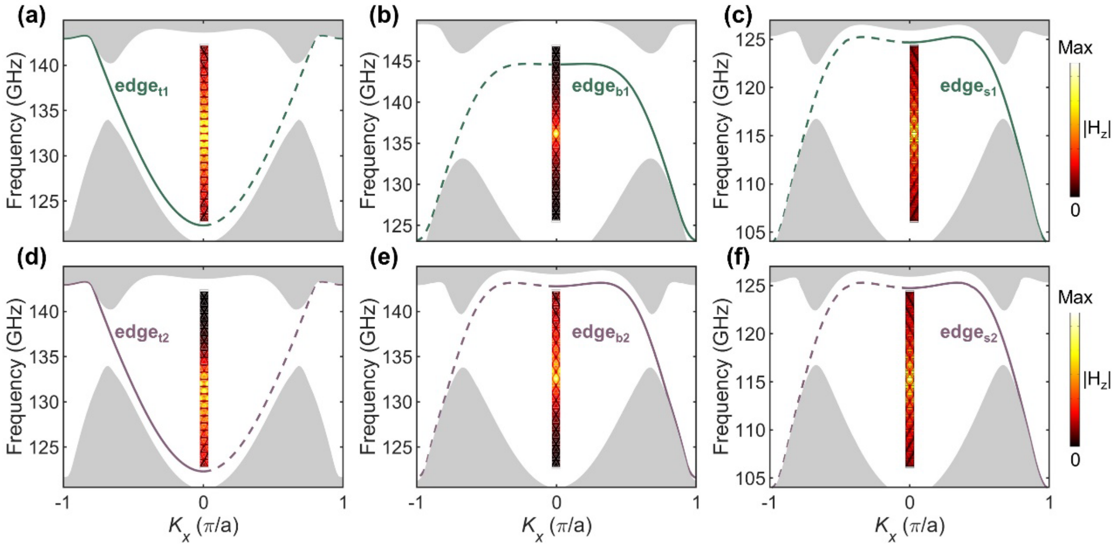

Figure S2. Dispersion of the reconfigurable edge states. (a-f), The Simulated dispersion of the valley kink states at $edge_{t1,2}$, $edge_{b1,2}$, and $edge_{s1,2}$. The inserts show the intensities of H_z near the domain walls. The solid curves in (a-f) represent valley kink states considered in subsequent simulations and experiments.

S3. THz on-chip Multichannel flexible multiplexing.

The operating frequency of flexible multiplexing chip can be correlated with the size of the bilayer photonic lattice. As shown in Fig. S3(a), by scaling the photonic lattice (keeping thickness unchanged), the operating channel can cover a continuous band from 110 GHz to 150 GHz. The relative size of the bandwidth of the two channels can also be adjusted by the two geometric parameters of Δl_t and Δl_b. This feature is conducive to the precise design of the operating frequency of each chip to meet the needs of flexible multiplexing of multiple channels. For example, as shown in Fig. S3(b), the scaling ratios of chip 1 to chip 5 are 0.88, 0.92, 0.96, 1, and 1.04 respectively. Therefore, the working spectrum of the 10 channels is continuous and does not overlap each other, which can support flexible multiplexing of terahertz broadband multichannel communications.

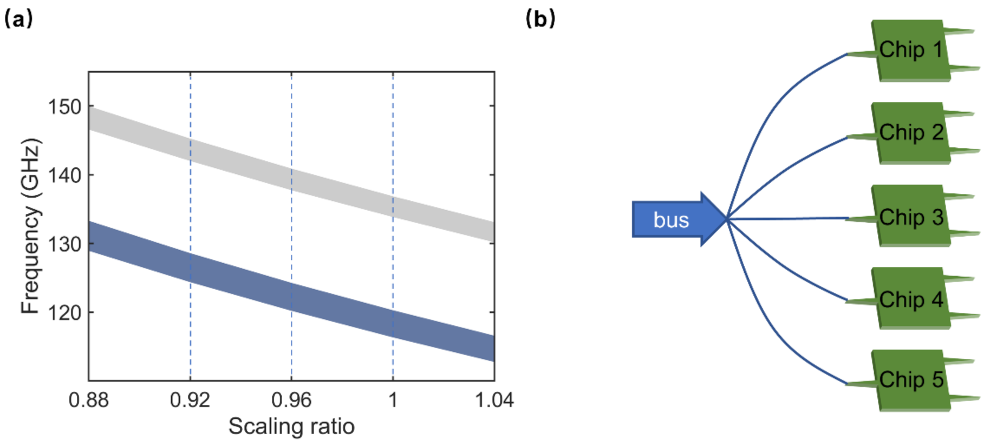

Figure S3. Broadband THz on-chip Multichannel flexible multiplexing architecture. (a), Operation spectrum covering from 110 to 150 GHz by scaling the two-dimensional lattice. (b), Multichip architecture for ultrawideband multichannel flexible multiplexing. The blue and gray region in Fig. S2(a) express the lower and higher band of the chip, respectively.

S4. THz communication measurement.

The optical spectrums of the laser-based signal source of the THz communication measurement system are shown in Figs. S4(a) and S4(d). When the chip operates in the coupled state, the S21 path is off while the S31 path is on at 120 GHz. Figs. S4(b) and S4(c) show the coupled-state electrical spectrum of port 2 and port 3, respectively. When the chip operates in the decoupled state, the S21 path is on while the S31 path is off at 130 GHz. Figs. S4(e) and S4(f) show the decoupled-state electrical spectrum of port 2 and port 3, respectively. Benefiting from the photonic band gap, the electrical spectrums show the isolation of 20 dB in both coupled state and decoupled state.

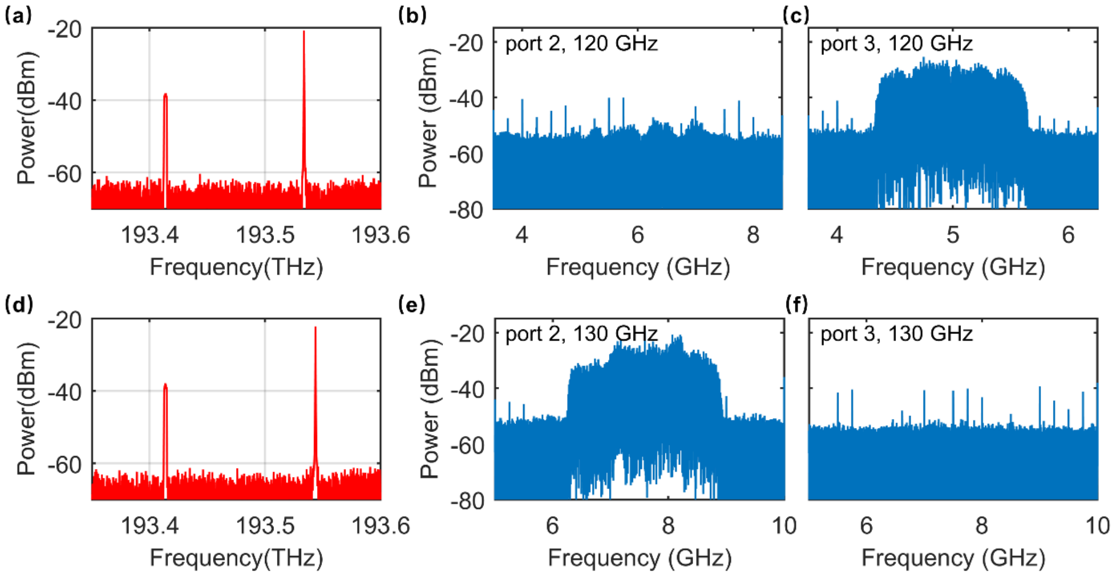

Figure S4. Optical spectrum and electrical spectrum of THz communication measurement. (a), The optical spectrum of the laser-based 120-GHz wave generation. (b, c), The electrical spectrum of S21 and S31 at coupled state around 120 GHz, respectively. (d), The optical spectrum of the laser-based 130-GHz wave generation. (e, f), The electrical spectrum of S21 and S31 at decoupled state around 130 GHz, respectively.

S5. Straight waveguide for normalization.

As shown in Fig. S5(a), the size of the straight waveguide used for normalization is 25.13 * 26.74 (mm). The lattice and edge state construction are consistent with *edge*$_{t1}$. The measured transmission of the straight waveguide is shown in Fig. S5(b). We also show the unnormalized S-parameters in the coupled and decoupled states in Figs. S5(c) and S5(d), respectively.

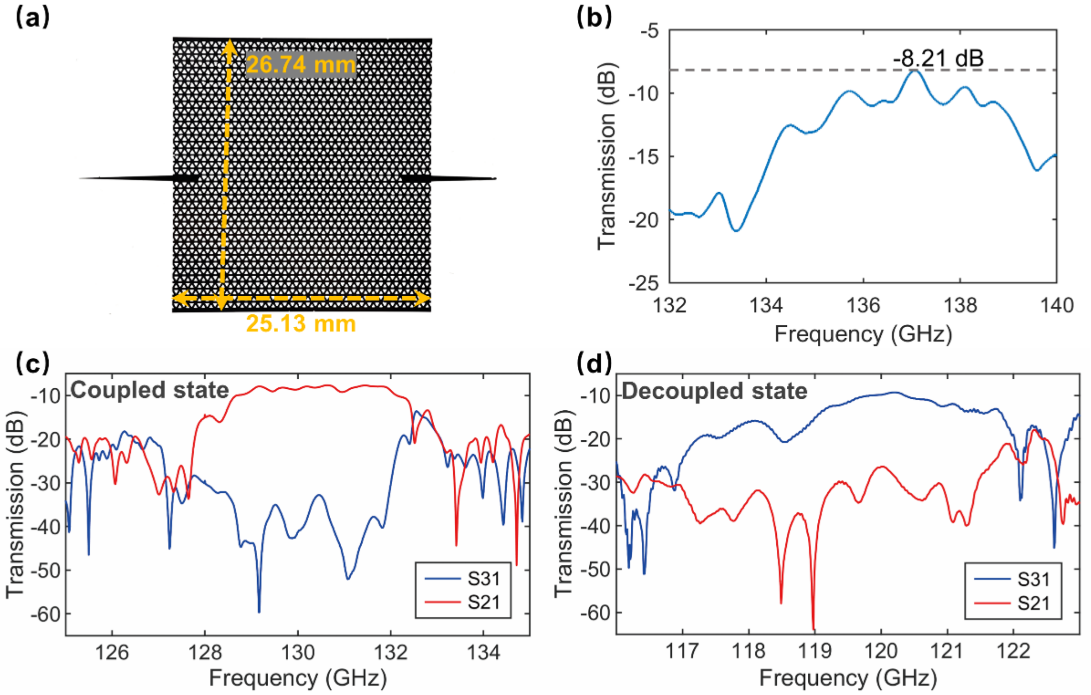

Figure S5. Straight waveguide for normalization and unnormalized S-parameters. (a), The optical photo and dimension of the straight waveguide for normalization. (b), The measured transmission of the straight waveguide for normalization. (c, d), Exact measured S-parameters of coupling state and decoupling state without normalization, respectively.

S6. Calculated transmission efficiency through integration of power.

In the practical experiment, we use a pair of silicon pins to connect the metal waveguide ports of VNA and the chip. It should be noted that, this conversion is not necessary in a full-VPC on-chip system. To investigate the realistic transmission loss inside our chip, we calculated the transmission efficiency inside the chip through the integration of power based on the results of the full-wave simulation. The relevant integration areas S1, S2 and S3 are highlighted in both layers by yellow dashed rectangles in Fig S6(a). We calculate the ratio of the integration of the power over the areas to eliminate the converting mismatch between the chip and the metal waveguide ports [S3, S4]. Figs. S6(b) and S6(c) show the transmission efficiency under the decoupled state and coupled state, respectively. These results demonstrate that the chip can transmit efficiently in both working states. Such a high transmission efficiency also indicates our chip can maintain an ultralow insertion loss in a silicon-based topological photonic on-chip system.

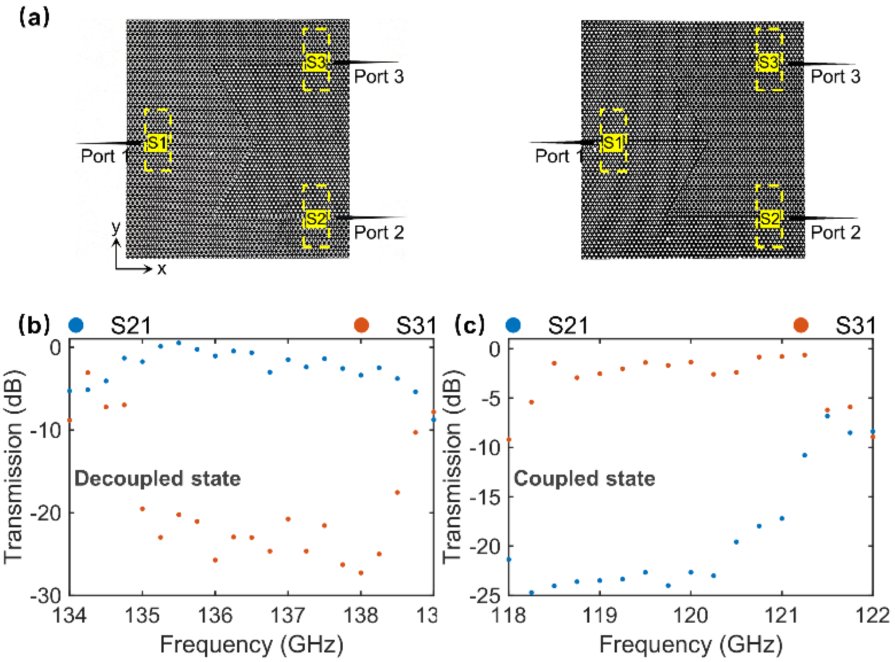

Figure S6. The in-chip transmission efficiency calculated through the integration of power. (a), Calculated integration areas S1, S2, and S3, which are highlighted by yellow dashed rectangular. (b), The decoupled state. (c), The coupled operating states.

S7. Modulation depth of the two reconfigurable channels.

As illustrated in Fig. S7(a) and S7(b), the modulation depth achieves as high as 18 dB and 21 dB for S31 path and S21 path, respectively.

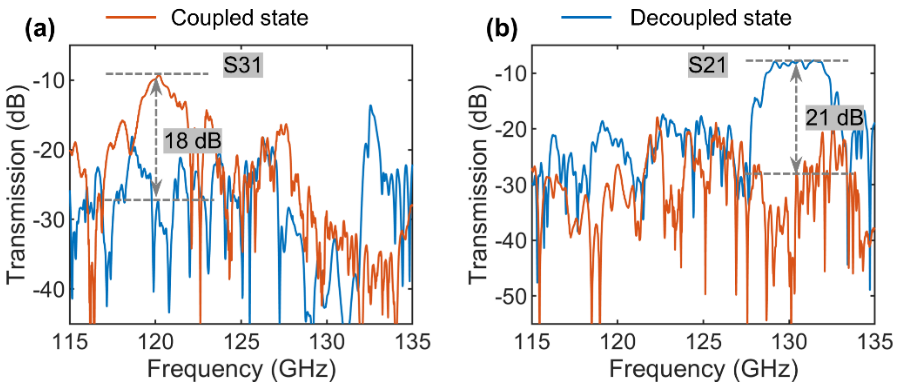

Figure S7. Modulation depth of the two reconfigurable channels.

Supplementary References

S1. P. R. Villeneuve, S. Fan, J. D. Joannopoulos, Phys. Rev. B **54**, 7837 (1996).

S2. T. Ozawa, H. M. Price, A. Amo, N. Goldman, M. Hafezi, L. Lu, M. C. Rechtsman, D. Schuster, J. Simon, O. Zilberberg, I. Carusotto, Rev. Mod. Phys. **91**, 015006 (2019).

S3. X. Jiang, C. Shi, Z. Li, S. Wang, Y. Wang, S. Yang, S.G. Louie, X. Zhang, Science **370**, 1447 (2020).

S4. W. Li, Q. Chen, Y. Sun, S. Han, X. Liu, Z. Mei, X. Xu, S. Fan, Z. Qian, H. Chen, Y. Yang, Adv. Optical Mater. **11**, 2300764 (2023).
